# Modulation in Techno-Functional, Textural Properties, In Vitro Starch Digestibility and Macromolecular–Structural Interactions of Pasta with Potato (*Solanum tuberosum* L.)

**DOI:** 10.3390/molecules27227835

**Published:** 2022-11-14

**Authors:** Savita Sharma, Nancy Malhotra, Arashdeep Singh, Rajan Sharma, Rubén Domínguez, José Manuel Lorenzo

**Affiliations:** 1 Department of Food Science and Technology, Punjab Agricultural University, Ludhiana 141001, Punjab, India; 2 Centro Tecnológico de La Carne de Galicia, ParqueTecnológico de Galicia, Adva. Galicia n° 4, San Cibrao das Viñas, 32900 Ourense, Spain; 3 Área de Tecnología de los Alimentos, Facultad de Ciencias de Ourense, Universidade de Vigo, 32004 Ourense, Spain

**Keywords:** pasta, potato, cooking quality, functional pasta, antioxidants, SEM, FTIR

## Abstract

The replacement of semolina with potato flour (PF) and potato mash (PM) at different levels was assessed for its effects on pasta quality. The results showed that the addition of PF and PM increased the pasting viscosity of the blends; in addition, PF enhanced the functional properties, while PM reduced them. The minimum cooking time decreased with PF and PM, while the PF pasta exhibited a higher cooking loss (5.02 to 10.44%) than the PM pasta, which exhibited a lower cooking loss. The pasta with PF and PM showed an increase in the total phenolic and flavonoid content, with reduced in vitro digestibility as confirmed by Fourier transform infrared spectroscopy. The PF pasta exhibited lower lightness and higher yellowness than the PM pasta, and its firmness and toughness also modulated owing to the complex interaction between potato starches and the gluten protein matrix, as evident from scanning electron microscopy. Sensory data revealed that pasta containing 30% PF and 16% PM was highly acceptable.

## 1. Introduction

Potato (*Solanum tuberosum* L.) is a nutritionally rich staple food, remarkable for its nutritional quality, vitamin C, vitamin B_6_, vitamin B_3_, complex carbohydrates, and minerals, such as phosphorus, magnesium, and potassium [1]. It has been greatly responsible for sustaining civilizations for generations. In addition to these basic nutrients, it is also known to contain notable antioxidants and phytochemicals, which contribute to its nutritional and health benefits, thereby making it highly desirable in the human diet [2,3]. The consumption of potatoes promotes gut microbiota, thereby regulating barrier properties and gut inflammation directly or indirectly [4]. The utilization of potato crops adds variety to our diet in addition to offering a number of desirable health and nutritional benefits, i.e., hypoglycemic, antioxidative, hypocholesterolemic, anti-obesity, anti-diabetic, and antimicrobial activities [5]. Despite its massive production, the surplus amount of potatoes in the glut season remains unexploited. In addition to this, the storage of potatoes results in various post-harvest losses. Evidently, the occurrence of storage crises has also resulted in increased instances of economic losses and frequent gluts in the food processing industry.

The processing of potatoes is the most sustainable and satisfactory means for its application in food products, which not only results in functionally compatible products but also solves the problem of the storage of bulky and perishable produce. The utilization of potato flour in snacks, bakery food, and noodles has contributed to various remarkable attributes, such as improved techno-functional characteristics and nutritional properties [5,6,7,8,9,10]. Mashed potatoes are used in the production of 3D-printed foods, as they are highly desirable ingredients to manufacture ready-to-eat products due to their fully gelatinized starch and rapid palate clearance, and they are considered mealy and warmly welcome throughout the world [11,12]. Potatoes are also used in the production of *gnocchi*, a typical Italian potato-based fresh pasta along with semolina or pseudo-cereals; it uses mashed potatoes that are either steamed cooked, pureed, or reconstituted [13,14]. Potato juice can also be used in the development of functional pasta owing to the high level of consumer acceptance of such pasta in comparison with that of classic pasta [15]. The addition of potato flour results in improvements in the nutritional, technological, rheological, and textural properties of steamed bread and noodles [16,17,18,19].

Due to the paradigm shift in the lifestyle of consumers due to globalization and urbanization, there is an increasing demand for health-based and convenient products [20]. The use of high-functionality ingredients to add value to pasta is presently a common strategy to deliver essential nutrients and other biologically active compounds which are generally absent in processed foods [5]. Pasta is a staple food in many countries and one of the most important foods consumed worldwide; it is made traditionally from durum wheat semolina and water, with other optional ingredients that have vitamins, minerals, dietary fiber, and phytochemicals in lower proportions [21]. The WHO and FDA recommend pasta as a good carrier for bioactive compounds and micro-nutrient delivery owing to its high acceptability among various population groups, affordability for all targeted classes, availability, easy preparation, low cost, and storage stability [22,23].

Though the functional and nutritional characteristics of pasta are important in terms of overall acceptability, textural characteristics may be more significant as they influence the preferences of consumers [24,25]. However, with ever-changing food preferences nowadays, consumers’ demand has moved from foods that provide energy to foods that give energy and have bioactive compounds and other nutritional benefits. The production and consumption of potatoes in pasta are encouraged as they increase the nutritional value of one’s diet [26]. Potato is a rich source of bioactive compounds and is easily available; as a result, an investigation has been planned to explore its utilization in the preparation of specialty pasta with enhanced nutritional, functional, textural, and antioxidant properties, thereby contributing to increased potato consumption by the general population.

## 2. Results and Discussion

### 2.1. Pasting Properties of the Blends

The incorporation of potato (flour and mash) in semolina significantly (*p* < 0.05) altered the pasting properties of the pasta blends. The inclusion of potato flour and potato mash led to a significant (*p* < 0.05) increase in the peak viscosity (PV), hold viscosity (HV), final viscosity (FV), setback viscosity (SV) and breakdown viscosity (BV) of the pasta blends in comparison to the control blend (Table 1). The control pasta blend had a pasting temperature (PT) of 94.2 °C, which decreased significantly (*p* < 0.05) from 93.7 to 91.1 °C and 93.1 to 91.1 °C as the levels of the incorporation of potato flour (20–40%) and potato mash (8–24%) increased, respectively, as a result of a dilution of the gluten network. The higher PT of the control pasta (94.2 °C) was due to fact that the high gelatinization temperature of the samples was correlated to their higher gluten content [16], and the reduction in the PT of the blends with potato flour and mash was due to the easier gelatinization of potato flour and mash owing to its higher starch content and lack of gluten protein, which rapidly absorbs water and gelatinizes [27].

Paste viscosity commonly refers to the water-holding capacity of starch granules and its measurement is an indication of the resistance of granules to swelling and shear performance [28]. Potato flour and mash significantly (*p* < 0.05) contributed to increased paste viscosity owing to the dilution of the gluten-starch network and the increased proportion of starch content (Table 1). The peak viscosity (PV) of the potato flour and potato mash blend increased significantly (*p* < 0.05) as the level of substitution increased in comparison to that of the control blend. This increased PV of the potato pasta blend was attributed to the early gelatinization of the starch and the increased amylose content as a result of the addition of potato flour and mash, thereby confirming the dominance of starch content in composite flour [16,29,30]. Furthermore, stronger intermolecular interactions in the potato flour and mash resulted in increased shear resistance and stability, and thus enhanced the PV. The inclusion of potato flour and mash also significantly increased the SV and FV compared to those of the control. This enhancement in the FV of the potato flour pasta blends may have been contributed by the amylose molecules further aggregating, resulting in increased gel hardness and paste stability [31]. However, the results showing a lower increase in the paste viscosity for potato mash in comparison to that of potato flour can be further correlated to starch re-association to some extent, resulting in significant alterations during the thermal process. In addition to this, the excessive water absorption of tubers might have interfered with the swelling of the starch, thereby delaying the process of gelatinization and resulting in a lower increase in the PV [29,32]. The control pasta had a SV of 1164 cP, which significantly (*p* < 0.05) decreased the incorporation of potato flour and varied from 1132 cP to 831 cP with increasing inclusion levels as a result of high amylose recrystallization, altered by the presence of potato proteins [33]. The pasting properties of the potato-flour-containing pasta, in other words the PV and HV, were positively correlated with the oil absorption capacity (OAC) (r = 0.981, r = 0.976, respectively) (*p* < 0.05), and the FV and BV were positively correlated with the water solubility index (WSI) (r = 0.952, r = 0.998, respectively) (*p* < 0.05), whereas the PT was negatively correlated with the water absorption capacity (WAC) and the WSI (r = 0.984, r = 0.964, respectively) (*p* < 0.05). The PT and SV of the potato-mash-containing pasta were positively correlated with the WSI (r = 0.965, r = 0.970, respectively) (*p* < 0.05). Studies by Sharma et al. [5] also reported a decrease in the pasting temperature and an increase in the pasting viscosity of flour blends with potato flour.

### 2.2. Functional Properties

The application of various ingredients in food products is significantly determined by their functional properties [34]. The functional properties of pasta blends formulated by incorporating potato flour and mash are presented in Table 2.

Functional properties, such as water absorption capacity, water solubility index. and oil absorption capacity, were estimated to enhance the utilization of potatoes in various food products. The water absorption capacity (WAC) of food products is an essential functional property and is closely related to the textural characteristics of the end products [35]. The control pasta had a WAC of 2.33 g/g. The WAC of pasta made by incorporating potato flour was found to be significantly different at higher inclusion levels (Table 2). Higher values of WAC have also been correlated to enhanced viscosity, which is desirable during the processing of gravies and soups [36]. The values ranged from 2.37 to 2.52 g/g. Contrasting results were obtained for the incorporation of potato mash; the WAC initially increased, followed by a non-significant decrease, and ranged from 2.32 to 2.61 g/g. This can be correlated to the hydrophilic nature of mash constituents compared to that of potato flour. Similar results were reported by Azizi et al. [36] and Sajilata et al. [37]. These studies reported a significant decline in WAC after boiling and relate it to the tendency of starch granules to undergo swelling, leading to water molecule imbibition during gelatinization. As a consequence of this, there was a subsequent increase in resistant starch, which prevents water absorption and justifies the reduction in the WAC. Thus, the variation in the WAC of tuber flour can be attributed to the difference in their varieties, as has been previously proposed by Jeddou et al. [38]. The control pasta had a WSI of 0.09 g/g, which increased significantly from 0.11 to 0.14 g/g as the level of the inclusion of potato flour increased. This can be explained by the dilution and weakening of the three-dimensional gluten protein, breakdown of its structural integrity, and disruption of the internal structure. This can also be correlated to the depolymerization of native starch during extrusion, as has been reported by Yadav et al. [39]. Wani et al. [40] reported that the increased water solubility and swelling power can be attributed to the high phosphate content of starch amylopectin. The phosphate groups might have influenced the adjoining chains, resulting in altered water hydration and solubility owing to the weakened bond formation within the crystalline part of the starch structure. These results were supported by a similar trend observed for the cooking loss in pasta containing potato mash at higher levels, in which the WSI decreased from 0.09 to 0.05 g/g and the cooking loss of the pasta containing potato mash decreased from 5.02 to 3.02% (Table 3). Singh et al. [41] reported lower WSI and WAC values for rice samples, further correlating it to the high lipid content of rice starch, owing to its ability to form long branched chains of amylopectin and amylose, thereby limiting the swelling power. The WSI and WAC of potato-flour-containing pasta was positively correlated with the water absorption (WA) (r = 0.973, r = 0.954) (*p* < 0.05), volume expansion (VE) (r = 0.961, r = 0.967) (*p* < 0.05), and cooking loss (CL) (r = 0.998, r = 0.983) (*p* < 0.05), whereas they were negatively correlated with the minimum cooking time (MCT) (r = 0.992, r = 0.969) (*p* < 0.05). In the pasta with the potato mash, the WSI was positively correlated with the MCT (r = 0.980) (*p* < 0.05), whereas it was negatively correlated with the VE (r = 0.999) (*p* < 0.05).

The oil absorption capacity (OAC) of the samples can be correlated to potatoes’ tendency to entrap oil owing to the capillary action, which can further contribute to the enhanced mouthfeel and flavor retention in the final products [42]. The incorporation of potato flour in pasta significantly influenced the OAC of the resulting pasta compared to that of the control pasta. The control pasta had an OAC of 1.86 g/g, which increased significantly and ranged from 1.98 to 2.02 g/g. This can be attributed to the alterations in the structure of the protein during extrusion, leading to increased lipophilic proteins and thus the OAC. Similar results were obtained for the incorporation of potato mash into pasta, although no significant difference was found at higher inclusion levels, with the values ranging from 1.89 to 1.92 g/g. The results were in agreement with Azizi et al. [36] and Onuegbu et al. [43]. Azizi et al. [36] also reported an increase in the OAC after boiling tubers, owing to the high level of oil uptake by the fiber from the potato peels. The high OAC of processed potato products is known to be beneficial for cultivars, owing to the better flavor-retention properties of the end products. 

### 2.3. Cooking Quality

The cooking quality of pasta is an essential characteristic when determining its consumer acceptability. It can be observed that potato pasta differs significantly on basis of its cooking quality (Table 3). Various cooking characteristics, such as the minimum cooking time—MCT (min), water absorption (%), volume expansion (mL/g), and cooking loss (%), were evaluated. The MCT can be regarded as the minimum time required to achieve the complete gelatinization of starch and reach the state of al dente, i.e., when pasta is cooked to be still firm when bitten. The MCT required by the semolina pasta was 6.30 min, which decreased significantly from 6 to 4.51 min as the levels of inclusion of potato flour increased from 20 to 40%. An analogous and noteworthy decrease was also reported in the case of pre-gelatinized pasta with potato mash, in which the values declined from 5.57 to 4.40 min with an increase in the substitution levels from 8 to 24%. This decline in the MCT of pasta could be justified by a considerable positive correlation with the pasting temperature (r = 0.84, *p* < 0.05) and a negative correlation with the water absorption (r = −0.81, *p* < 0.05). It indicates that the incorporation of potato flour and potato mash resulted in subtler temperatures of gelatinization along with the superior availability of water molecules, which were ultimately responsible for a significant drop in the MCT of the resulting pasta. Sharma et al. [5] also confirmed an analogous relationship between potato flour and the optimum cooking time of potato/cereal pasta.

Interestingly, the pasting temperature was negatively correlated with the water absorption (r = −0.87, *p* < 0.05), which indicates that a complete gelatinization of starch granules is favored at low temperatures owing to the superior absorption and improved uptake of water. These conditions were also responsible for the notable up-surge in the volume expansion of both the cooked potato flour (1.92 to 2.32 mL/g) and potato-mash-incorporated (1.85 to 2.01 mL/g) pasta samples. A significant positive correlation (r = 0.91, *p* < 0.05) was also noted among the water absorption and volume expansion of the cooked pasta samples. The outcomes of the present investigation were in complete agreement with the study conducted by Saleh et al. [44], which evaluated the comparable effects of sweet potato flour incorporation on the functional and cooking behavior of wheat pasta. 

Approaching the caliber of water absorption by the potato-flour-incorporated pasta samples, the values increased significantly (*p* < 0.05) and ranged from 221.22 to 271.51% as the substitution level increased from 20 to 40%. In contrast, for potato-mash-incorporated pasta samples, the water absorption ranged from 241.56 to 247.43 with an increase in the substitution levels from 8 to 24%. At the same time, the increase in water absorption was not that pronounced in the case of pre-gelatinized potato-mash-incorporated pasta. The chief reason behind this could be the delayed and restricted absorption of water by pre-gelatinized starches [44]. Sharma et al. [5] also established a comparable inter-association between the techno-functional properties of potato flour with the cooking characteristics of the resulting paste. The remarkable influence of potato flour was chiefly due to three factors: (1) the morphological distinctiveness of the starch granules in comparison to that of wheat semolina; (2) inconsistency in the starch and protein interactions; and (3) irregularity in the resulting 3D matrix [44,45]. The enhanced water absorption behavior of potato flour- and mash-incorporated pasta could be attributed to the higher water affinity of the potato flour due to having a higher fiber content, which caused a physical disruption of the native protein–starch matrix to yield a course for the diffusion of water molecules [5].

For pasta incorporated with potato flour, the cooking loss was significantly altered at higher inclusion levels (Table 3). The control pasta had a cooking loss value of 5.02%. The cooking loss ranged from 6.06 to 10.44 % when the inclusion ranges from 20 to 40%. In pasta, a stable starch–gluten matrix can remarkably reduce cooking loss. However, the addition of potatoes led to the dilution of the gluten protein, breakdown of its structural integrity, and disruption of the internal structure in pasta as a result of the higher leaching of amylose, as was reported by Bartova et al. [6]. The results were also in agreement with the water solubility index of pasta. This can be further correlated to the disruption of inter- and intramolecular hydrogen bonds in the starch network, thereby facilitating increased starch solubility [39]. This trend was, however, reversed in the incorporation of potato mash, in which the addition of mash at various levels led to non-significant variations in the cooking loss values. Potato mash pasta had a cooking loss content ranging from 4.35% to 3.02% at varying inclusion levels. This might be due to the presence of a large portion of gelatinized starch. The strong and stable internal bonds formed as a result of gelatinization prevent the leaching out of amylose due to the better adhesion of gelatinized mash and thereby have a lower solubility during cooking [46]. 

### 2.4. Bioactive Constituents and Antioxidant Activity

The incorporation of potato flour and mash led to a significant increase in the bioactive constituents and antioxidant activity of the resulting potato pasta (Table 4). The incorporation of potato flour in pasta led to a significant increase in the total phenol content of the control pasta, which had a TPC of 1.13 mg GAE/g. The pasta prepared by incorporating potato flour had a TPC range of 2.28 to 2.79 mg GAE/g. This may be due to the increased levels of phenolic compounds in tubers compared to semolina, as potato contains higher amounts of polyphenolic compounds, mainly chlorogenic acid [47]. However, it was observed that in the case of the mash with values ranging from 1.28 to 1.71 mg GAE/g, the TPC was lower.

**Figure 1:**
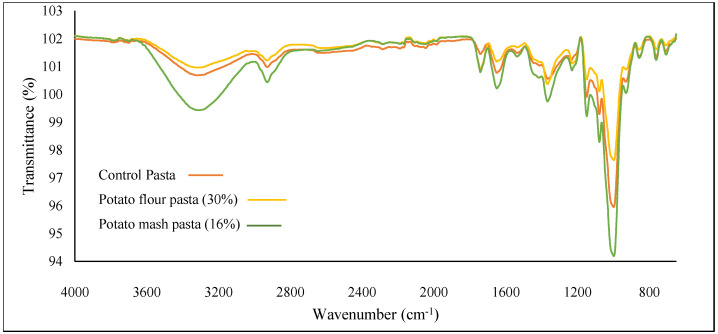
FTIR spectra of control pasta, potato flour pasta, and potato mash pasta.

Mattila and Hellstrom [48] reported a considerable reduction in the phenolic content in cooked potatoes in comparison to that of uncooked potatoes. This variation in the phenolic content of pasta may be due to the heat treatment during the mash preparation. This can be also attributed to the combination of processing losses due to the degradation from the thermal treatment, leaching into the water, isomerization, and action of polyphenol oxidase [49]. The higher phenolic content of potato pasta in comparison to the control pasta can be associated with an increase in the antioxidant extractability from the potato matrix, owing to the structural and textural changes in cooking. The presence of phenols was also confirmed by the characteristic peaks observed at the 1662.7 cm^−1^ region in FTIR spectroscopy (Figure 1). Flavonoids are the most common group of plant phenolic compounds which are known to differ significantly with respect to their antioxidant activity. Potato flavonoid extracts have also been reported to depict high scavenging activities [50]. The control pasta had a flavonoid content of 10.18 mg quercetin/100 g, and the incorporation of potato flour led to a significant increase in the flavonoid content of the resulting pasta. The pasta incorporating potato flour and mash had a flavonoid content range from 15.41 to 19.15 mg quercetin/100 g and 11.01 to 13.92 mg quercetin/100 g, respectively. Perla et al. [51] reported the flavonoid content of potatoes to be more than 30 mg/100 g fresh weight. Potato is a potential source of numerous phenolic compounds, the majority of which are localized in the peel and adjoining tissues of tubers [52].

The DPPH radical scavenging activity of pasta made by the addition of potato differed significantly in comparison to that of the control pasta (Table 4). Antioxidants in potatoes contribute to the defense against free-radical-mediated and oxidative reactions. The control pasta had an antioxidant activity of 19.11% RSA, which increased from 25.75 to 28.19% RSA and from 19.81% to 21.62% RSA as the levels of incorporation of potato flour and mash increased. The presence of phytochemicals, such as polyphenols, carotenoids, ascorbic acid, flavonoids, and α-linoleic acid, in the potato accounts for the enhanced antioxidant activity of potato-flour- and potato-mash-incorporated pasta [52]. Jayanty et al. [53] reported potatoes to be an excellent source of antioxidants despite the compositional and structural changes that take place during cooking. The antioxidant activity (DPPH) of potato-flour- and potato-mash-incorporated pasta was positively correlated with the total phenolic content (TPC) (r = 0.996, r = 0.992) (*p* < 0.05) and total flavonoid content (TFC) (r = 0.973, r = 0.998) (*p* < 0.05). Compositional alterations in antioxidants further result in variations in the antioxidant activity in potato tubers after cooking and process methods that lead to the increased retention of antioxidant compounds, contributing to a high level of antioxidant activity [53]. The decline in the antioxidant activity values of potato mash pasta in comparison to those of potato flour pasta can be attributed to the processing conditions. Similar to vitamin C, other phenolic antioxidants are hydrophilic and tend to leach from tissues by processing in water. The boiling of tubers also significantly affects their antioxidant activity, which can be supported by the water-soluble nature of phenols, thereby justifying the lower antioxidant activity of potato mash pasta in comparison to potato flour pasta [51].

### 2.5. In Vitro Starch Digestibility

In recent times, pasta has gained immense popularity owing to its low glycemic index which correlates to the structural characteristics of pasta, leading to a compact starch–protein network, further inhibiting the action of amylolytic enzymes, and decreasing the glucose release rate in the human body. The in vitro starch digestibility of potato pasta differed significantly from that of the control pasta, as can be seen in Table 4. The starch digestibility of the control pasta was observed to be higher than that of the potato pasta. The uncooked and cooked control pastas had a starch digestibility of 56.92 g maltose/100 g flour and 67.44 g maltose/100 g flour, respectively. The value of starch digestibility of the potato flour pasta was lower compared to that of the control pasta, and with an increase in the levels of potato flour, the values for in vitro starch digestibility decreased, ranging from 36.25 to 38.03 g maltose/100 g flour. This can be due to the large particle size of potato starch granules in comparison to cereal starch, which might have limited the availability of the surface area required by amylase [54]. The study also reported that the variation might have also been the result of a partial disruption of the starch chain structure during the preparation of flour. Furthermore, the presence of bioactive compounds, such as phenolics, and flavonoids in the potato flour restricts the enzymatic activity of amylases, and thus the samples exhibit a lower level of starch digestibility. Cao et al. [55] illustrated that steamed bread produced with potato pulp as a substitute for wheat flour showed a decrease in the glycemic index as a result of reduced starch digestibility. An increase in the starch digestibility of cooked pasta was observed in comparison to that of uncooked pasta, owing to the alteration in the organized starch structure further facilitating the attack of enzymes. The values of starch digestibility of the cooked potato flour pasta ranged from 69.20 to 67.44 g maltose/100 g flour, while for the potato mash pasta, it ranged from 65.23 to 59.15 g maltose/100 g flour.

The value of the starch digestibility of the potato mash pasta was also lower compared to that of the control pasta, and with an increase in the levels of potato mash, the values for in vitro starch digestibility decreased, ranging from 50.76 to 40.48 g maltose/100 g flour. When the pasta was made with potato mash, the presence of gelatinized starch resulted in the complex network between potato gelatinized starch, wheat starch, and gluten protein, which could prevent the further leaching of amylose. Additionally, this could form a barrier for the amylases to enter and attack the starch, which leads to the low in vitro starch digestibility observed in potato mash pasta [56]. Starch digestibility can also be correlated to a combination of factors, such as the amylose content, starch source, degree of crystallinity, granule size, etc. The variation in the starch digestibility of potato mash pasta is possibly due to the boiling of tubers followed by cooling, resulting in the retrogradation of amylose in the mash. The results were in agreement with Raigond et al. [57], who reported a similar result in tuber products under conditions in which boiling was followed by cooling for mash preparation, leading to the formation of resistant starch as a result of physio-chemical modifications. The lower starch digestibility of potato mash pasta is also indicative of its high resistant starch content [36]. The progressive decline in the starch digestibility of potato mash pasta compared to potato flour pasta also correlates to the presence of retrograded starch that is formed as a result of the cooling of mash post-preparation (boiling) leading to pasta that is rich in resistant starch. The esterified phosphate group connected to the glucose residue of the starch prevents amylase from acting, therefore the full hydrolysis of potato starch with the phosphate group yields phosphoryl-oligosaccharides, and potato starch that has high levels of phosphate groups has a poorer enzyme digestibility [58]; as a result of this, pasta with potato flour and mash exhibits lower digestibility. 

### 2.6. Color Characteristics

Color is one of the most impactful factors when it comes to determining the overall quality of the product because it affects the consumer acceptance of the product, which in turn affects the market value of the product [59,60]. Product color indicates the nature and quality of ingredients used in making pasta products. The control pasta showed the highest L* (lightness) value (57.08), which decreased significantly from 46.48 to 42.36 as the level of incorporation of potato flour increased from 20 to 40%, while the L* value of the pasta incorporated with potato mash decreased significantly from 54.49 to 48.46 as the level of incorporation of potato mash increased from 8 to 24%. The L* value of the potato-flour- and mash-incorporated pasta was negatively correlated with the TPC (r = 0.994 and r = 0.971) (*p* < 0.05) and TFC (r = 0.990 and r = 0.994) (*p* < 0.05) of the uncooked pasta. The incorporation of potato flour and mash significantly increased the redness (a*) and yellowness (b*) values of the pasta (Table 5). The incorporation of potato flour enhanced the redness and yellowness values to a greater extent (0.20–2.65 and 9.92–14.87), in comparison to pasta prepared with potato mash, in which there was a minor enhancement in the redness values (−1.38 to −1.14) and yellowness values (5.58–5.71). In this case, a bright yellow color is considered a positive attribute for pasta which increases consumers’ preference for these products. Studies by Alessandrini et al. [14], and Kowalczewski et al. [15] also reported an increase in the redness value of the pasta and *gnocchi* with the inclusion level of potato flour and potato juice. The reduction in the lightness/brightness (L*) and the higher yellowness value of potato-flour- and mash-incorporated pasta were due to the darker color of the flour and mash in comparison to that of the semolina, owing to the presence of pigments, bioactive compounds, and flavonoids; these result in the formation of pigments, such as melanin, caused by the non-enzymatic browning reaction of pasta due to the interaction of non-wheat components with wheat components, such as polyphenol oxidase [15]. Furthermore, the lowest L* and highest a* and b* values of the potato flour pasta in comparison to the potato mash pasta were due to the presence of a higher number of pigments, such as polyphenols and carotenoids, and also the vitamin C in the potato powder, which resulted in the formation of melanoid compounds during the production or the polyphenol oxidase reaction with the phenolic components to a greater extent [47,52]. Studies by Kosovic et al. [61] and Zhang et al. [62], also reported a decrease in L* values and an increase in a* redness values and b* values with an increase in the level of chestnut flour in pasta and sweet potato flour in noodles. The minor changes in the L*, a*, and b* values of the pasta with the potato mash in comparison to the potato flour were attributed to the gelatinization of the starch during the mash preparation, which resulted in the inhibition of the polyphenol oxidase reaction and also reduced the polyphenol and antioxidant content, as evident from Table 4. Studies by Galvez et al. [63] reported a significant relationship between the degree of the gelatinization of starch during processing and the clarity of the noodles. Alessandrini et al. [14] also reported similar changes in the lightness values of pasta made from steam-cooked potato, potato puree, and reconstituted-potato-based *gnocchi*. The higher changes in the yellowness value and lightness values of pasta with potato flour could also be due to the yellow color of potato flour compared to semolina and also due to the heat-sensitive nature and higher sugar content of potato powder, which when heated for an extended period, makes a complex with a protein to form a Maillard reaction product [64,65].

These results can also be seen clearly from the data for the total color change presented in Table 5. When the value of ΔE is more than 3.5–5 units, an experienced observer can clearly identify a color divergence between two or more tested subjects; however, when values are less than these units, consumers could not see the apparent difference [66]. The results showed that the addition of potato flour resulted in a higher total color change or ΔE values from 17.02 to 20.10 as the level of incorporation increased from 20 to 40%; however, in the case of potato mash, the value for ΔE ranges from 11.22 to 13.46 as the level of incorporation increased from 8 to 24%, and thus, it is possible to state that the samples of pasta fortified with both flour and mash varied from the control samples to the point in which a color change was evident to the human eye (ΔE > 11). Studies by Kowalczewski et al. [15] also reported similar results for the ΔE values of pasta with fresh potato juice and potato juice flour, exhibiting ΔE values of 14.14 to 15.89. 

### 2.7. Textural Pproperties

The texture is an important attribute in the consumer acceptability of pasta, and higher firmness and toughness are parameters for good-quality pasta products [59]. The results of toughness and firmness for the uncooked and cooked pasta with potato flour showed an overall decrease and with the potato mash showed an overall increase in comparison to the control pasta (Table 6). The addition of potato flour (20–40%) into semolina decreased the firmness of pasta, ranging from 2.16 to 2.85 kg/s in uncooked pasta and 0.10–0.17 kg/s in cooked pasta; however, the inclusion of potato mash (16–24%) increased the firmness of the uncooked pasta from 5.15 to 5.85 and the cooked pasta from 0.28 to 0.39 in comparison to the control (3.74 for the uncooked and 0.26 for the cooked pasta). The lower firmness and toughness values of the cooked pasta in comparison to those of the uncooked pasta were due to water absorption during cooking [14]. Firmness is a true reflection of intermolecular forces between the swelling of starch molecules within a protein matrix. This decrease was attributed to a decrease in the protein content of the pasta and the disulphide bond of the pasta with potato flour. Firmness is related to protein content, which induces a strong protein network among the pasta samples [67]. Therefore, a reduction in protein and an increase in starch content weakens the internal structure of dry pasta, which may lead to the formation of cracks or discontinuities inside the pasta strands and weaken the pasta’s structure. Consequently, the dilution in the gluten network increases the likelihood of being crushed [13,68]. The dilution of the gluten network due to the addition of starch (with a high affinity for water) with the inclusion of potato flour decreased the availability of water for the development of the gluten matrix. Nawaz et al. [64] in their report also showed that the quality of noodles deteriorated as the level the potato flour substitution increased.

The increased firmness of the pasta with potato mash could be due to the interaction between the potato and wheat flour components, as the presence of more gelatinized starch in the potato flour helped the blends to absorb water, resulting in increased adhesion and a higher level of firmness [69]. Meanwhile, on the other hand, the toughness values for the uncooked and cooked pasta increased as the levels of potato flour and potato mash also increased, as a result of the increased potato starch molecular bonding with the semolina starch, in which the amylose molecules were rearranged [70]. Similar results were also reported by Kowalczewski et al. [15] and they also observed that the form in which potatoes were added to pasta influences the textural quality of the final product. 

### 2.8. FTIR Molecular Interactions

FTIR spectroscopy is an advanced technique to comprehend constitutional functional groups based on their vibration patterns. As shown in Figure 1, the patterns of absorption bands were similar for the control pasta and the pasta incorporated with potato flour and mash, implying the presence of identical compounds. The FTIR spectra showed characteristic peaks of C-H, C-O, and C=C bonds for all the samples, which indicates the presence of phenolics molecules in all samples. The sharp peak shown at around 1000 cm^−1^ basically corresponds to the stretching of C-O-H vibrations and was related to the amorphous state of the starch granules [71]. The amide I and amide III regions ranged from 1500 to 1800 cm^−1^ and from 1200 to 1400 cm^−1^, respectively. The secondary structure of the proteins was mainly implied by the amide I zone comprising α-helix (1650–1664 cm^−1^), β-sheet (1613–1640 cm^−1^ and1680–1695 cm^−1^), β-turn (1664–1680 cm^−1^), and random coil (1640–1650 cm^−1^) [72]. The transmittance peak observed at around 1500 cm^−1^ signifies the presence of amide II band, resulting from vibrations of the N-H bond with CN stretching. The peak observed around 1650 cm^−1^ mainly showed the higher intensity of the amide I band region, which was due to the increase in the protein content of the WGP-incorporated pasta [73]. 

The changes in macronutrients, i.e., starch and protein, due to the incorporation of potato flour and mash led to changes in carbohydrate zone (750–1200 cm^−1^ fingerprint region) and amide zones as observed by changes in transmittance. Furthermore, the absorbance peaks observed around regions 2350, 2360, and 2950 cm^−1^ signify stretching vibrations of the CN and N–H bond. Higher changes were observed in potato mash due to starch gelatinization and protein denaturation during pressure cooking. The peaks observed between the regions of 3200–3650 cm^−1^ were attributed to the stretching vibration of the O–H bond and the peaks ranges between 3700 and 3900 cm^−1^ signified the presence of the O–H group. The intense band around 3300 cm^−1^ represented O-H stretching vibration, especially due to phenolic groups. Higher absorption (reduced transmittance) was observed in pasta incorporated with potato flour which reiterated the increase in bioactive constituents and antioxidant activity. Potato mash exhibited increased transmittance, which was also observed for steamed potato flour by Zhang et al. [74]. Modifications around these bands implied changes in the control and specialty pasta, which led to the exhibition of varied constitutional, bioactive, and functional properties. 

### 2.9. Microstructure of Pasta

**Figure 2:**
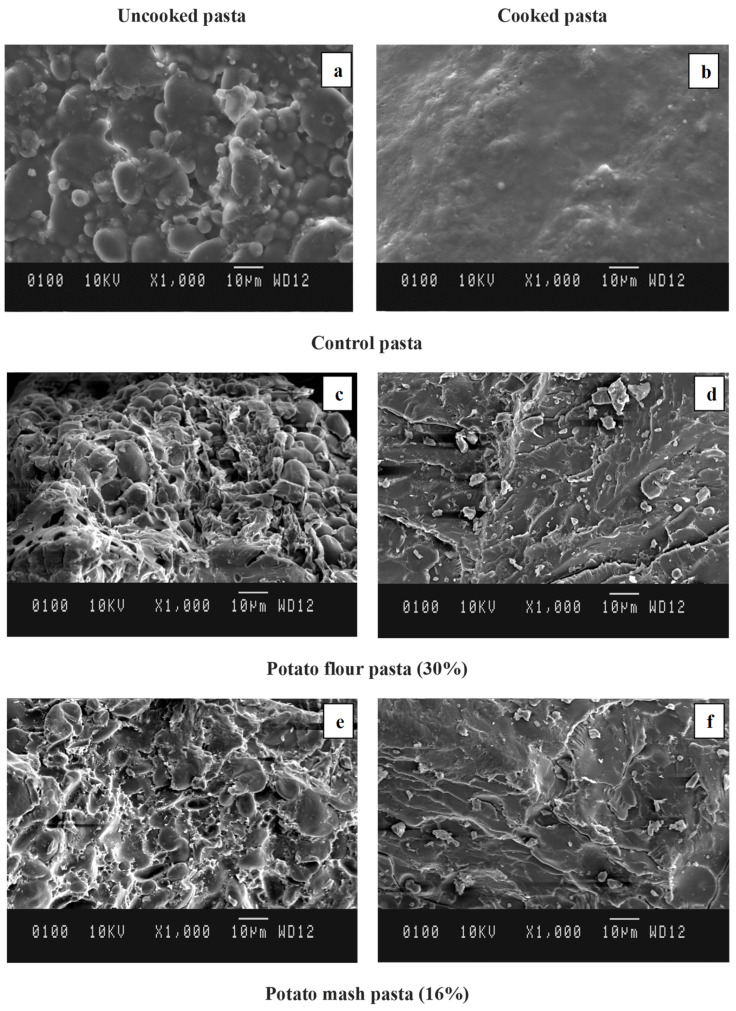
Scanning electron microscopic images of uncooked (**a**,**c**,**e**) and cooked (**b**,**d**,**f**) control, potato flour and potato mash pasta.

A scanning electron microscope (SEM) creates an image by focusing an electron beam over the surface of the sample to examine its microstructure. The electrons present in the beam interact with the sample and produce various signals that are used to obtain information topography and the composition of the surface of the sample. The microstructure of the uncooked and cooked pasta samples was investigated using a SEM, which provided a clear picture of the changes in starch and protein granules because of the addition of the potato flour and mash in the pasta, along with the modifications observed before and after cooking this pasta (Figure 2a–f). In the uncooked samples, the presence of starch granules of different shapes and sizes showed the predominance of wheat starch with its spherical-lenticular shape. In Figure 2c,e, the presence of large oval elliptical granules confirms the presence of potato starch in the form of flour and mash [13]. In all the cases, the uncooked pasta samples exhibited visible starch granules, whereas, in cooked pasta samples, the outer surface was smooth due to the complete embedding of the starch granules in the protein matrix. During the cooking of the pasta, the gelatinization of starch and the diffusion of amylose into the continuous space occurred, which in turn was attributed to the expansion of the pasta during cooking, leading to stress on the protein network [75]. The uncooked pasta containing potato flour exhibited starch granules of varied sizes. However, the uncooked pasta incorporated with potato mash exhibited eroded starch granules due to the gelatinization for starch, which was present as highly ordered fragments with compact structures and irregular surfaces due to the aggregation phenomenon [74]. This justified the low in vitro starch digestibility of the uncooked pasta containing potato mash. However, the loose protein starch network upon cooking indicated an enhanced entry of α-amylase, which was also supported by the high in vitro starch digestibility of the cooked pasta samples.

The cooked control pasta in Figure 2b exhibited a highly smooth surface, whereas the pasta containing potato flour in Figure 2d and mash in Figure 2f exhibited a disrupted continuous surface. The presence of gluten protein formed an emulsifying network and a film over the starch granules, which was evident in the cooked control pasta (Figure 2b). This film formation of proteins over the starch granules limits the water diffusion, swelling, and gelatinization [13], which can also be evident from the higher cooking time and lower swelling volume of the control pasta. Furthermore, the presence of gelatinized starch in the pasta from potato mash imbibed the wheat starch granules and protein well (Figure 2e), and these formed a complex structure that increased the hardness, which is also evident in the present study. In the control pasta after cooking, the granules were swollen and diffused, showing a closer visible starch–protein network, which was not evident in the potato pasta. Studies by Kowalczewski et al. [15] also reported that the effects of fortification on the microstructure were dependent on the form in which the potato was introduced, as the microstructure of pasta samples depends on the interaction between starch and the protein fraction. They reported that the addition of potato juice powder resulted in a loose microstructure of the pasta, which was also observed in the present study. Gelatinized starch was formed during the potato mash preparation, which also acted as a hydrocolloid binding agent, and when the pasta was made with potato mash, the presence of gelatinized starch resulted in the complex network between the potato gelatinized starch, wheat starch, and gluten protein, which restricted the leaching of swollen starch into the cooking water during cooking [56], thereby reducing the cooking loss, as evident from the data presented in Table 3. The formation of the viscous gelatinized starch–starch protein layer on the surface of cooked potato mash pasta could prevent the further leaching of amylose and this also would form a barrier against the amylases to prevent them entering and attacking starch, which would lead to the low in vitro starch digestibility observed in the potato mash pasta.

### 2.10. Sensory Evaluation

Pasta formulated by the addition of potato (flour and mash) was evaluated on basis of its sensory quality using a nine-point hedonic scale (Table 7).

Significant variations were found among the potato flour pasta in regard to flavor, texture, and overall acceptability compared to those of the control pasta. The control pasta had an overall acceptability score of 8.7. The potato flour pasta with 20% and 30% inclusion levels was found to be non-significantly different compared to the control pasta with an overall acceptability of 8.2 and 8.1, respectively. The inclusion level of 40% had a significantly lower acceptability compared to that of the other inclusion levels, in terms of the texture, flavor, and appearance of pasta, which can be due to the high water-absorption capacity of potato flour. Pasta at higher levels of inclusion, upon cooking, was found to be loose and sticky. Based on the overall acceptability score, potato flour incorporation at 30% was well received. In the case of incorporation of potato mash in pasta, inclusion levels of 8% and 16% were found to be equally acceptable. Lower inclusion levels of potato mash pasta (8% and 16%) in pasta were found to be non-significantly different from the control pasta with an overall acceptability of 8.30 for both. The overall acceptability of the potato mash pasta was found to be significantly lower at 24%. Potato mash pasta containing 16% mash had a higher acceptability in the context of its flavor perception. The flavor of the pasta is an essential sensory attribute in order to determine its acceptability. It can therefore be concluded that the incorporation of flour and mash beyond 30% and 16% led to an overall decline in the acceptability of pasta. 

## 3. Materials and Methods

### 3.1. Preparation of Potato Flour and Potato Mash

Potato tubers (*Solanum tuberosum*, var. *Kufri Pukhraj*) were procured from the Department of Vegetable Science, Punjab Agricultural University, Ludhiana, Punjab. The tubers were cleaned under running water to remove adhered dirt before further processing. Potatoes and sweet potatoes were initially peeled manually, using a mechanical peeler, and hand-trimmed with a knife. Slices of 2–3 mm of thickness were cut from the peeled tubers manually followed by soaking in 0.5% potassium metabisulphite solution for 15 min to prevent enzymatic browning and later dried at 50 °C in an NSW-154 hot air cabinet tray drier for 7–8 h and later ground into flour and sieved using a 60-mesh sieve (BIS) corresponding to 250 µm particle size. The prepared flours were stored in pearl pet jars and stored under ambient conditions for further analysis and product preparation. Thoroughly washed and cleaned potatoes tubers were pressure-cooked using autoclave (15 min at 121 °C, 15 psi). Processed potatoes tubers were then peeled and mashed by passing then through a wire strainer. The potato flour (20, 30, and 40%) and potato mash (8, 16, and 24%) (based on primary trials, data not shown) were then mixed with semolina at varying proportions before cold extrusion for product development.

### 3.2. Preparation of Potato-Flour- and Potato-Mash-Incorporated Functional Pasta

The pasta blends were mixed with the optimum amount of water for 10–12 min in the mixing chamber of the pasta extruder (La Monferrina Masoero Arturo and C.S.N.C., Vespucci, Asti, Italy) to uniformly hydrate the particles. The dough was extruded using an adjustable macaroni shape (die No. 82) with a corrugated opening of 1.5 mm. The macaroni shape pasta was cut into a desirable length of 1.0 cm, by adjusting the rotator cutter speed. The extruded pasta was dried at 45–50 °C for 5–6 h using a drying oven (Naarang Scientifics, New Delhi, India) which was packed in polypropylene (PP) bags and stored at 4 °C till further analysis.

### 3.3. Pasting and Functional Properties of Pasta Blends

#### 3.3.1. Pasting Properties

The pasting properties of the WGP-incorporated pasta blends was evaluated using Rapid Visco analyzer (RVA) (Newport Scientific, Warrie Wood, Australia) [60]. Briefly, 3.5 g sample (14% moisture basis) was taken and mixed with distilled water (25 mL). The programmed heating and cooling cycle of the short temperature profile (profile 1) (13 min) was conducted for 10 sec at 960 rpm and for another test at 160 rpm. Then the sample was equilibrated at a temperature of 50 °C for 60 sec and subsequently heated to 95 °C with a 12.2 °C/min heating rate. After that, it was kept at 95 °C for 2.5 min. Then, the sample was cooled to 50 °C at 12.2 °C/min, and held at this temperature for 2.1 min.

#### 3.3.2. Functional Properties

Water absorption capacity (WAC) and water solubility index (WSI) of the blends were determined according to the method described by Singh et al. [35] and Surasani et al. [60] with slight modification. Sample (3.0 g) was taken in a pre-weighed 50.0 mL centrifuge tube; to this, 30.0 mL deionized water was added and kept for 30 min with intermittent shaking every 5 min. It was then centrifuged (Sorvall ST 16R, Thermo Fisher Scientific, Dreieich, Germany) at 3000 rpm for 15 min. The supernatant was transferred in a pre-weighed Petri dish and kept for drying at 100 °C for 5–6 h. Thereafter, weights of dry solids and gel were noted. The WAC and WSI were calculated using the following Equations: (1) and (2).

(1) $$\mathrm{WAC}=\frac{\mathrm{Weight}\;\mathrm{of}\;\mathrm{gel}\;\mathrm{afer}\;\mathrm{removal}\;\mathrm{of}\;\mathrm{supernatant}\left(g\right)}{\mathrm{Weight}\;\mathrm{of}\;\mathrm{sample}\left(g\right)}\times 100$$

(2) $$\mathrm{WSI}=\frac{\mathrm{Weight}\;\mathrm{of}\;\mathrm{dry}\;\mathrm{solids}\left(g\right)}{\mathrm{Weight}\;\mathrm{of}\;\mathrm{sample}\;\left(g\right)}\;\times 100$$

#### 3.3.3. Oil Absorption Capacity

Sample (2.0 g) was added with 20.0 mL refined sunflower oil in a pre-weighed 50 mL centrifuge tube. It was then incubated for 30 min with intermittent shaking every 5 min. The content was centrifuged at 3000 rpm for 15 min and the supernatant was collected in a pre-weighed Petri dish [35]. The oil absorption capacity (AC) was calculated using following equation (Equation (3)).

(3) $$\mathrm{OAC}=\frac{\mathrm{Weight}\;\mathrm{of}\;\mathrm{gel}\;\mathrm{after}\;\mathrm{removal}\;\mathrm{of}\;\mathrm{supernatant}\;(g)}{\mathrm{Weight}\;\mathrm{of}\;\mathrm{sample}\;\left(g\right)}\;\times 100$$

### 3.4. Cooking Quality

The cooking quality of pasta in terms of minimum cooking time (MCT), water absorption (WA), volume expansion, and cooking loss were calculated using the standard methods of AACC [76]. The minimum cooking time was the time required for complete disappearance of the white core, thereby indicating complete gelatinization of starch. Water absorption was calculated by estimating the increase in weight during cooking and calculated as the percentage of water absorption. The volume expansion is noted by calculating the difference in the volume expansion of water before and after cooking the pasta. Cooking loss is the loss of gruel solids in the cooked water calculated by drying the cooked water to obtain solid residue.

### 3.5. Sensory Evaluation of Functional Pasta

Sensory evaluation of the cooked pasta samples was conducted by a panel of 75 members age ranging from 20 to 55 years, from the Department of Food Science and Technology, Department of Food and Nutrition, Punjab Agricultural University, Ludhiana, India. The panelists were prior informed about the product and after their consent they were asked to evaluate the pasta samples. Samples were evaluated for appearance, color, flavor, texture, taste, appearance, and overall acceptability using 9-point hedonic scale ranging from extremely liked (9) to extremely disliked (1).

### 3.6. Antioxidant Properties and Bioactive Components of Functional Pasta

The antioxidant activity of pasta was measured using DPPH (2,2-diphenyl-1-picrylhydrazyl) as per the method described by Sharma et al. [42]. Total phenolics and flavonoids were calculated with the methodology described by Kataria et al. [72]. The methanolic extract of pasta samples prepared using acidified methanol was used to evaluate phenolic and flavonoid contents and results were expressed as mg gallic acid and catechin equivalents, respectively.

### 3.7. In Vitro Starch Digestibility

In vitro starch digestibility of pasta (raw and cooked) was determined by estimating the amount of maltose formed by using the dinitro salicylic acid (DNS) method as per Singh et al. [28]. Briefly, 100 mg finely ground sample and 100 mg of pancreatin-alpha amylase enzyme was taken in a conical flask along with 20 mL phosphate buffer (pH 6.9) and incubated at 37 °C for 2 h. After incubation, the contents were filtered and initial drops were discarded. A volume of 1 mL of filtrate was then taken in test tube to which 2 mL DNS was added and test tube was then boiled in a water bath for 5 min followed by cooling. Then, 50 mL volume was made up in a volumetric flask and absorbance was taken at 540 nm. The standard curve of maltose was used to estimate mg of maltose released corresponding to the absorbance of sample.

### 3.8. Color Characteristics of Functional Pasta

Color characteristics of uncooked and cooked pasta (L*, a*, b*) were calculated using Color Flex meter (Hunter Lab Color Flex, Hunter Associates Inc., Reston, VA, USA) [59]. The L*, a* and b* values were noted and total change in color (∆E) due to enrichment was calculated using the following equation (Equation (4)).

(4) $$\Delta E=\sqrt{{\left(\Delta L*\right)}^{2}+{\left(\Delta a*\right)}^{2}+{\left(\Delta b*\right)}^{2}}$$

### 3.9. Textural Attributes of Pasta

TA-XT plus texture analyzer (Stable Micro Systems, Godalming, Surrey GU7 1YL, UK) was utilized to evaluate the properties, such as firmness and toughness, of the uncooked and cooked pasta. The test was carried out under compression mode by cutting the sample with a knife blade to a distance of 10 mm with a speed of 1 mm s^−1^. Trigger force of 10.0 g was used with pre- and post-test speeds of 2 mm s^−1^ as per the modified method used by Singh et al. [59]. Briefly, system consists of a small Perspex blade with a fitting that is located directly in the loadcell. The sample is placed on the heavy-duty platform, the base of the texture analyser, and text was performed.

### 3.10. Fourier Transform Infrared Spectroscopy (FTIR) Analysis of Functional Pasta

The analysis of pasta with consideration of the influence of the addition of potato flour and potato mash on the functional group of pasta was carried out using a Fourier transfer infrared (FTIR) spectrophotometer (Alpha Bruker, Billerica, MA, USA). The sample of pasta was kept on the FTIR sample holder and infrared spectra were noticed at wavelengths ranging between 500 and 4000 cm^−1^.

### 3.11. Statistical Analysis

The data reported in all the tables are averages of triplicate observations. The data were inspected by one-way analysis of variance (ANOVA) and means were compared using post hoc Tukey’s test at (*p* < 0.05). The data were subjected to statistical analysis using SPSS Statistical Software version 18.0 (SPSS Inc., Chicago, IL, USA).

## 4. Conclusions

Potato can successfully be utilized as a non-conventional ingredient in the development of functional pasta owing to its bioactive compounds with potential health benefits. The utilization of potato in flour (30%) and mash (16%) forms for the development of specialty pasta has resulted in products with enhanced functional and biochemical constituents without altering the quality characteristics of specialty pasta. The resulting pasta exhibited favorable starch digestibility, with a promising cooking quality and overall acceptability owing to its higher water absorption and favorable yellow color. Furthermore, the utilization of potato not only helps to reduce post-harvest losses of potato but also helps to enhance the functionality of pasta with non-conventional ingredients.

## Figures and Tables

**Table 1:** Effect of replacing potato powder and mash with semolina on the pasting of pasta blends.

Blends	Levels (%)	Pasting Temperature (°C)	Peak Viscosity (mPa⋅s)	Hold Viscosity (mPa⋅s)	Final Viscosity (mPa⋅s)	Breakdown Viscosity (mPa⋅s)	Setback Viscosity (mPa⋅s)
Semolina	-	94.2 ± 0.3 ^a^	648 ± 09 ^f^	538 ± 08 ^f^	1722 ± 17 ^f^	90 ± 03 ^g^	1164 ± 18 ^a^
Potato flour	20	93.7 ± 0.3 ^b^	1144 ± 32 ^d^	951 ± 12 ^c^	1977 ± 19 ^c^	193 ± 06 ^e^	1132 ± 11 ^b^
	30	92.3 ± 0.2 ^d^	1339 ± 13 ^b^	1146 ± 11 ^b^	2183 ± 11 ^b^	243 ± 10 ^d^	1069 ± 21 ^d^
	40	91.1 ± 0.3 ^e^	1514 ± 07 ^a^	1167 ± 03 ^a^	2236 ± 10 ^a^	327 ± 16 ^c^	831 ± 19 ^f^
Potato mash	08	93.1 ± 0.1 ^c^	774 ± 09 ^e^	642 ± 12 ^e^	1814 ± 09 ^e^	132 ± 08 ^f^	1102 ± 15 ^c^
	16	91.9 ± 0.1 ^d^	1201 ± 11 ^c^	848 ± 03 ^d^	1876 ± 05 ^d^	347 ± 04 ^b^	1097 ±11 ^c^
	24	91.1 ± 0.2 ^e^	1219 ± 07 ^c^	854 ± 05 ^d^	1891 ± 16 ^d^	371 ± 07 ^a^	1028 ± 08 ^e^

Values are expressed as Mean ± SD (n = 3). ^a–g^ Means with different superscripts in a column differ significantly (*p* < 0.05) from each other.

**Table 2:** Effect of replacing potato powder and mash with semolina on the functional properties of pasta blends.

Blends	Levels (%)	Water Absorption Capacity (g/g)	Water Solubility Index (g/g)	Oil Absorption Capacity (g/g)
Semolina	-	2.33 ± 0.03 ^c^	0.09 ± 0.01 ^cd^	1.86 ± 0.02 ^d^
Potato flour pasta	20	2.37 ± 0.01 ^c^	0.11 ± 0.02 ^bc^	1.98 ± 0.01 ^b^
	30	2.42 ± 0.02 ^bc^	0.12 ± 0.01 ^ab^	1.99 ± 0.01 ^ab^
	40	2.52 ± 0.03 ^b^	0.14 ± 0.01 ^a^	2.02 ± 0.04 ^a^
Potato mash pasta	08	2.61 ± 0.07 ^a^	0.08 ± 0.01 ^d^	1.92 ± 0.02 ^c^
	16	2.39 ± 0.14 ^c^	0.07 ± 0.02 ^de^	1.89 ± 0.01 ^cd^
	24	2.32 ± 0.01 ^c^	0.05 ± 0.02 ^e^	1.89 ± 0.01 ^cd^

Values are expressed as Mean ± SD (n = 3). ^a–e^ Means with different superscripts in a column differ significantly (*p* < 0.05) from each other.

**Table 3:** Effect of replacing potato powder and mash with semolina on the cooking quality of the pasta.

Type of Pasta	Levels (%)	Minimum Cooking Time (Min)	Water Absorption (%)	Volume Expansion (mL/g)	Cooking Loss (%)
Control	-	6.30 ± 0.04 ^a^	212.95 ± 2.11 ^f^	1.79 ± 0.01 ^f^	5.02 ± 0.09 ^c^
Potato flour pasta	20	6.00 ± 0.02 ^b^	221.22 ± 1.34 ^e^	1.92 ± 0.04 ^d^	6.06 ± 1.67 ^bc^
	30	5.29 ± 0.07 ^d^	251.36 ± 1.28 ^b^	2.18 ± 0.01 ^b^	7.34 ± 1.98 ^b^
	40	4.51 ± 0.01 ^f^	271.51 ± 2.83 ^a^	2.32 ± 0.05 ^a^	10.44 ± 0.27 ^a^
Potato mash pasta	08	5.57 ± 0.04 ^c^	241.26 ± 2.21 ^d^	1.85 ± 0.02 ^e^	4.35 ± 0.14 ^cd^
	16	5.05 ± 0.02 ^e^	243.56 ± 3.05 ^cd^	1.91 ± 0.10 ^d^	3.71 ± 0.47 ^d^
	24	4.40 ± 0.03 ^g^	247.43 ± 2.05 ^bc^	2.01 ± 0.04 ^c^	3.02 ± 0.11 ^d^

Values are expressed as Mean ± SD (n = 3). ^a–g^ Means with different superscripts in a column differ significantly (*p* < 0.05) from each other.

**Table 4:** Bioactive constituents, antioxidant activity, and in vitro starch digestibility of potato powder and mash pasta.

Type of Pasta	Levels (%)	Total Phenol Content (mg GAE/g)	Flavonoids (mg QE/100 g)	Antioxidant Activity (%DPPH RAS)	Starch Digestibility	
					Uncooked	Cooked
Control	-	1.13 ± 0.06 ^g^	10.18 ± 0.82 ^g^	19.11 ± 0.04 ^f^	56.92 ± 0.98 ^a^	71.25 ± 0.03 ^a^
Potato flour pasta	20	2.28 ± 0.08 ^c^	15.41 ± 0.26 ^c^	25.75 ± 0.13 ^c^	38.03 ± 0.50 ^e^	69.20 ± 0.24 ^b^
	30	2.51 ± 0.13 ^b^	18.01 ± 0.11 ^b^	26.12 ± 0.09 ^b^	37.03 ± 0.18 ^ef^	68.16 ± 0.12 ^c^
	40	2.79 ± 0.12 ^a^	19.15 ± 0.06 ^a^	28.19 ± 0.06 ^a^	36.25 ± 0.52 ^f^	67.44 ± 0.08 ^d^
Potato mash pasta	08	1.28 ± 0.02 ^f^	11.01 ± 0.03 ^f^	19.81 ± 0.25 ^f^	50.76 ± 1.43 ^b^	65.23 ± 0.23 ^e^
	16	1.42 ± 0.03 ^e^	12.54 ± 0.17 ^e^	20.66 ± 0.13 ^e^	45.03 ± 1.38 ^c^	61.46 ± 0.17 ^f^
	24	1.71 ± 0.01 ^d^	13.92 ± 0.27 ^d^	21.62 ± 0.28 ^d^	40.88 ± 0.81 ^d^	59.15 ± 0.12 ^g^

Values are expressed as Mean ± SD (n = 3). ^a–g^ Means with different superscripts in a column differ significantly (*p* < 0.05) from each other.

**Table 5:** Effect of replacing potato powder and mash with semolina on the color characteristics of pasta.

Type of Pasta	Levels (%)	Color Characteristics			
		L*	a*	b*	(ΔE)
Control	-	57.08 ± 0.42 ^a^	−1.57 ± 0.32 ^f^	4.43 ± 0.15 ^f^	-
Potato flour pasta	20	46.48 ± 0.61 ^de^	0.20 ± 0.44 ^c^	9.92 ± 0.09 ^c^	17.02 ± 0.08 ^c^
	30	43.15 ± 1.15 ^e^	0.89 ± 0.08 ^b^	10.54 ± 0.11 ^b^	18.13 ± 0.42 ^b^
	40	42.36 ± 0.18 ^e^	2.65 ± 0.14 ^a^	14.87 ± 0.07 ^a^	20.10 ± 0.61 ^a^
Potato mash pasta	8	54.49 ± 0.49 ^b^	−1.38 ± 1.14 ^e^	5.58 ± 0.02 ^d^	11.22 ± 0.26 ^f^
	16	50.84 ± 0.42 ^c^	−1.17 ± 0.3 1^d^	5.60 ± 0.15 ^de^	12.69 ± 0.43 ^e^
	24	48.46 ± 0.28 ^d^	−1.14 ± 0.07 ^d^	5.71 ± 0.21 ^e^	13.46 ± 0.18 ^d^

Values are expressed as Mean ± SD (n = 3). ^a–f^ Means with different superscripts in a column differ significantly (*p* < 0.05) from each other. L (0-100)*-blackness to lightness and a*- + value for red and -ve value for green, b*- + value for blue and -ve value for yellow.

**Table 6:** Effect of replacing potato powder and mash with semolina on the textural attributes of pasta.

Type of Pasta	Levels (%)	Uncooked		Cooked	
		Firmness	Toughness	Firmness	Toughness
Control	-	3.74 ± 0.13 ^c^	0.94 ± 0.50 ^f^	0.26 ± 0.02 ^c^	0.29 ± 0.01 ^e^
Potato flour pasta	20	2.16 ± 0.24 ^e^	1.08 ± 0.39 ^e^	0.10 ± 0.01 ^f^	0.31 ± 0.01 ^d^
	30	2.48 ± 0.70 ^de^	1.56 ± 0.03 ^c^	0.16 ± 0.01 ^de^	0.35 ± 0.03 ^c^
	40	2.85 ± 0.72 ^d^	1.75 ± 0.66 ^b^	0.17 ± 0.01 ^d^	0.47 ± 0.02 ^ab^
Potato mash pasta	8	5.15 ± 0.90 ^b^	1.10 ± 0.06 ^e^	0.28 ± 0.01 ^c^	0.33 ± 0.03 ^cd^
	16	5.73 ± 0.28 ^a^	1.38 ± 0.12 ^d^	0.35 ± 0.03 ^ab^	0.44 ± 0.02^b^
	24	5.83 ± 0.47 ^a^	2.08 ± 0.98 ^a^	0.39 ± 0.04 ^a^	0.48 ± 0.01 ^a^

Values are expressed as Mean ± SD (n = 3). ^a–f^ Means with different superscripts in a column differ significantly (*p* < 0.05) from each other.

**Table 7:** Effect of replacing potato powder and mash with semolina on the sensory characteristics * of pasta.

Type of Pasta	Levels (%)	Appearance	Flavor	Texture	Overall Acceptability
Control	-	8.7 ± 0.46 ^a^	8.8 ± 0.40 ^a^	8.7 ± 0.46 ^a^	8.7 ± 0.46 ^a^
Potato flour pasta	20	8.5 ± 0.50 ^a^	8.0 ± 0.45 ^ab^	8.2 ± 0.40 ^bc^	8.2 ± 0.40 ^a^
	30	8.4 ± 0.49 ^a^	8.2 ± 0.60 ^ab^	7.9 ± 0.70 ^bc^	8.1 ± 0.30 ^a^
	40	7.7 ± 0.64 ^b^	6.5 ± 0.46 ^c^	7.4 ± 0.49 ^d^	6.8 ± 0.75 ^b^
Potato mash pasta	08	8.5 ± 0.50 ^a^	8.3 ± 0.46 ^ab^	8.4 ± 0.49 ^ab^	8.3 ± 0.46 ^a^
	16	8.3 ± 0.64 ^a^	8.5 ± 0.50 ^a^	8.3 ± 0.46 ^ab^	8.3 ± 0.46 ^a^
	24	8.2 ± 0.75 ^ab^	7.5 ± 0.67 ^b^	7.8 ± 0.60 ^cd^	7.0 ± 0.89 ^b^

* Sensory score out of 9.0. Values are expressed as Mean ± SD (n = 3). ^a–d^ Means with different superscripts in a column differ significantly (*p* < 0.05) from each other.

## Data Availability

All data are available from the authors.

## Sample Availability

Samples of the compounds are not available from authors.
